# Stevens–Johnson syndrome induced by toripalimab in a previously EGFR-TKI-treated advanced lung adenocarcinoma patient harboring *EGFR* mutations 19 del/T790M/C797S in *trans* and *cis*: a case report

**DOI:** 10.3389/fphar.2023.1131703

**Published:** 2023-11-14

**Authors:** Yang Chen, Hanhan Hong, Shujun Bao, Hao Tang

**Affiliations:** Department of Respiratory and Critical Care Medicine, Changzheng Hospital, Naval Medical University, Shanghai, China

**Keywords:** Stevens–Johnson syndrome, adverse reaction, toripalimab, non-small-cell lung cancer, EGFR tyrosine kinase inhibitor

## Abstract

**Background:** The treatment paradigm for advanced non-small-cell lung cancer (NSCLC) is rapidly changing. Epidermal growth factor receptor tyrosine kinase inhibitors (EGFR-TKIs) and anti-programmed death-1 (PD-1) antibodies have increasingly been incorporated into routine care for nearly all patients with NSCLC. Toripalimab was recently approved as the first-line treatment for advanced non-squamous NSCLC in combination with chemotherapy. Stevens–Johnson syndrome (SJS) is a rare but potentially fatal complication of TKI and anti-PD-1 therapy. We reported a case of SJS after sequential use of EGFR-TKIs and toripalimab in an NSCLC patient with *EGFR* mutations 19 del/T790M/C797S in *trans* and *cis*.

**Case presentation:** A 58-year-old man with stage IV NSCLC received gefitinib because next-generation sequencing (NGS) revealed an *EGFR* 19del, followed by osimertinib and pemetrexed with the emergence of *EGFR* T790M. Four *EGFR* mutations 19 del/T790M/C797S in *trans* and *cis* were detected after osimertinib resistance. The combination of toripalimab and docetaxel was administered as a third-line treatment. The patient developed SJS at 21 days, and toripalimab was discontinued. After treatment with methylprednisolone and prednisolone, the skin toxicity of the patient gradually decreased and eventually disappeared. The patient received osimertinib and anlotinib after recovery, and SJS has not recurred. The ongoing treatment is still effective and results in stable disease.

**Conclusion:** We reported the first case of SJS induced by toripalimab in a patient with lung adenocarcinoma harboring multiple *EGFR* mutations. The TKI treatment after SJS was well tolerated and effective.

## Introduction

Lung adenocarcinoma is one of the most common types of non-small-cell lung cancer (NSCLC). The treatment paradigm for advanced NSCLC is rapidly changing. Epidermal growth factor receptor (EGFR) is the most common driver genes of lung cancer, and EGFR tyrosine kinase inhibitors (TKIs), such as gefitinib, osimertinib, and anlotinib, dramatically improve the clinical outcomes of *EGFR* mutant lung cancers (Han et al., 2018; Soria et al., 2018; Hosomi et al., 2020). At the same time, immune checkpoint inhibitors (ICIs), such as anti-programmed death-ligand-1 (PD-(L)1) monoclonal antibodies, have increasingly been incorporated into routine care for nearly all patients with NSCLC. Pembrolizumab was approved as the front-line therapy with or without chemotherapy in patients with metastatic NSCLC (Reck et al., 2016). Nivolumab plus ipilimumab was approved as a first-line treatment for NSCLC patients by the Food and Drug Administration (Hellmann et al., 2019). Toripalimab, an anti-PD-1 antibody, significantly improved both progression-free survival (PFS) and overall survival (OS) with chemotherapy in patients with advanced NSCLC with a manageable safety profile (Wang et al., 2023).

However, cutaneous eruptions are one of the most common immune-related adverse events, including lichenoid reactions, eczema, and vitiligo (Hwang et al., 2016), most of which are mild. Stevens–Johnson syndrome (SJS) is a rare but life-threatening cutaneous adverse reaction, mainly elicited by exposure to certain drugs including EGFR-TKIs and ICIs (Chen et al., 2018). There is growing concern that the combination of PD-(L)1 and EGFR-TKIs may be associated with an increased risk of toxicity. It is reported that PD-(L)1 blockade followed by osimertinib is associated with severe immune-related adverse events (Schoenfeld et al., 2019). There were no reports of SJS in a patient treated with toripalimab and EGFR-TKIs. Here, we reported the first case of SJS induced by toripalimab in a previously EGFR-TKI-treated advanced lung adenocarcinoma patient harboring multiple *EGFR* mutations.

## Case presentation

**FIGURE 1:**
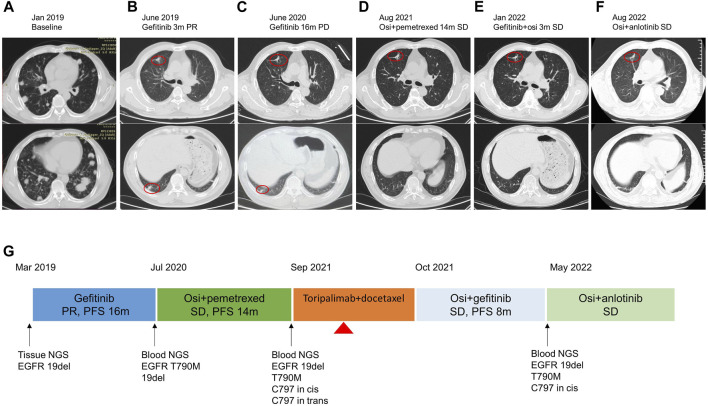
Treatment timeline of the patient. **(A–F)** The chest CT images at each time point. The red circle indicated the tumor lesion. **(G)** Flow diagram of the clinical course of the patient. Osi, osimertinib; PR, partial response; PD, progressive disease; SD, stable disease; PFS, progression-free survival. The red triangle indicates the SJS onset on day 12 since treatment with toripalimab plus docetaxel.

A 58-year-old male non-smoker presented to our hospital complaining of persistent pain in the lower back in January 2019. He had no existing physical health issues and no special underlying diseases. The family medical history was unremarkable. The enhanced computerized tomography (CT) scan of the chest and lumbar spine revealed multiple nodules in both the lungs and spinal lesions (Figure 1A). A CT-guided percutaneous needle biopsy was performed. The pathological examination showed lung adenocarcinoma. Together, these results suggested the clinical stage was classified as cT4N3M1, stage IV (TNM classification seventh edition). The patient performance status (PS) was 1. Next-generation sequencing (NGS) identified *EGFR* exon 19 deletion (19 del) with a mutant allele frequency (MAF) of 60.2%. The patient was begun on gefitinib 250 mg once daily in March 2019. Partial response (PR) was achieved with 3 months’ treatment based on the Response Evaluation Criteria in Solid Tumors (RECIST) 1.1 (Figure 1B), and the PFS was 16 m. New lesions were seen in the left lower lobe (Figure 1C), and gefitinib was discontinued. Blood-based NGS detected *EGFR* T790M (MAF 3.4%) and the retention of *EGFR* 19 del (MAF 5.99%). Osimertinib (oral) and pemetrexed (0.9 g, iv, q3w) were administered in July 2020, and stable disease (SD) was achieved with a PFS of 14 m. The patient complained of pain in the lower back accompanied by numbness in the lower leg in August 2021. The spine magnetic resonance imaging (MRI) revealed more spinal lesions, and the chest CT scan showed the lung nodules were stable (Figure 1D).

NGS targeting eight core lung cancer driver genes (Lung Cure, Burning Rock Biotech, Guangzhou, China) was performed on blood samples. *EGFR* mutations 19 del/T790M/C797S in *trans* and *cis* were detected, with MAF of 1.7%, 0.44%, 0.16%, and 0.14%, respectively. In a phase-II trial, toripalimab plus chemotherapy showed promising anti-tumor activity as the second-line setting in patients with *EGFR*-mutant NSCLC (Jiang et al., 2021). In September 2021, the patient received toripalimab 240 mg and docetaxel 120 mg as the third-line therapy.

**FIGURE 2:**
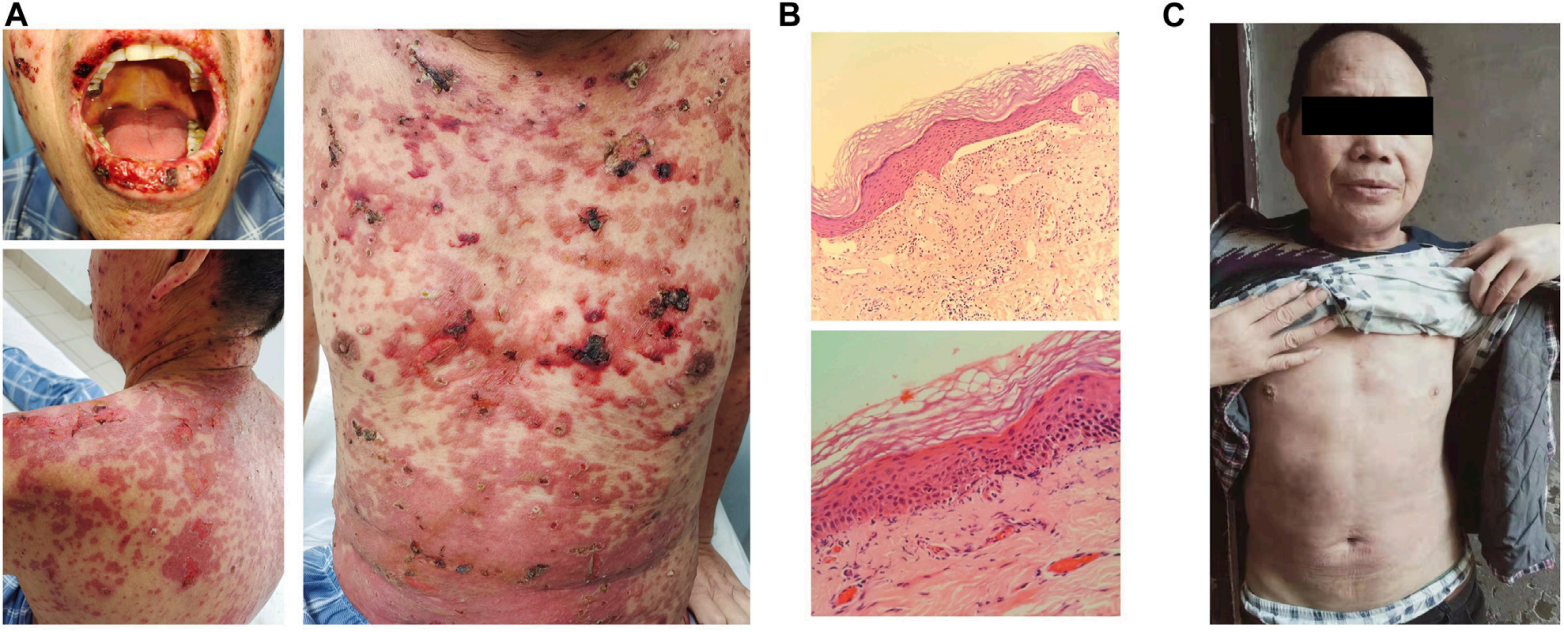
Diagnosis and treatment of Stevens–Johnson syndrome. **(A)** Erosions were seen on the mouth, face, and body trunk after 24 days of toripalimab treatment. **(B)** Hematoxylin and eosin staining of skin biopsy. Original magnifications, the upper panel × 40 and the lower panel × 100. **(C)** Reduction in diffuse erythema at 6 weeks of steroid therapy.

The patient began to develop oral ulcers and scattered rash on the 12th day since treatment with toripalimab plus docetaxel, and the rash gradually worsened. He had not received treatment for the skin reactions before visiting our hospital on day 24. He presented with multiple macules and vesicles, and detachment of the epidermis on the mucous membranes of the mouth, face, and body trunk (Figure 2A). He had no fever, and the PS was 1. Routine blood examinations were normal. Bacterial cultures from blood, urine, and sputum revealed no evidence of bacterial infection. Skin biopsy showed a sub-epidermal cell poor blister and perivascular infiltrate of lymphocytes (Figure 2B). Throughout the course of the disease, there were no other organ function abnormalities. He had normal levels of alanine aminotransferase, aspartate aminotransferase, creatinine, urea nitrogen, cardiac enzymes, or brain natriuretic peptide. The patient was diagnosed as SJS with a severity-of-illness score for toxic epidermal necrolysis (SCORTEN) as 4 (Supplementary Table S1). In terms of pharmacogenetic assessments, we performed human leukocyte antigen (HLA) typing using NGS, which revealed HLA-A*24:02, HLA-A*11:01, HLA-B*40:01, HLA-B*15:01, HLA-C*04:01, and HLA-C*03:04.

Toripalimab and docetaxel were discontinued immediately. A dermatologist was consulted for the diagnosis of skin symptoms. The patient presented with diffuse erythema, and vesicles and ulcerations on extremities, the trunk, oral cavity, throat, nose, eyelids, and genitalia, which were accompanied by skin detachment and tissue necrosis. These manifestations were consistent with SJS. We also checked serum levels of several autoantibodies, including anti-BM antibody, anti-AD antibody, and anti-EC antibody, all of which were negative. Taking into account the clinical and pathological manifestations and the medication history, we arrived at the diagnosis of SJS. The patient was treated with 100 mg/day of methylprednisolone on 10 October 2021 for 1 week, followed by 90 mg/day of prednisolone for 3 weeks. Prednisolone was tapered off and eventually discontinued after 2 months. The patient recovered from SJS after steroid therapy (Figure 2C). During this period, the patient took medicine at home, and the local broken surface was disinfected with iodine and covered with dry gauze to keep the wound dry. He did not experience any concurrent infection.

Afterward, the patient received spine stereotactic body radiotherapy (SBRT) to treat spinal metastases. The combination of gefitinib and osimertinib was administered between October 2021 and May 2022, and SD was achieved with a PFS of 8 m (Figure 1E). A follow-up CT scan revealed stable lung nodules but more spinal lesions, which led to pathological bone fracture and paraplegia. NGS targeting 520 cancer-related genes (OncoScreen Plus, Burning Rock Biotech, Guangzhou, China) was performed on blood samples in May 2022 and revealed mutations of *EGFR* 19 del/T790M/C797S in *cis*, with MAF of 18.9%, 3.53%, and 3.06%. The patient received osimertinib 80 mg and anlotinib 12 mg in May 2022. A CT scan showed the shrinkage of the lung tumors (Figure 1F) and stable lesions of the spine after 2 months. The patient declined the surgery for spinal metastasis because of financial concerns. He is still treated with osimertinib and anlotinib, and SJS has not recurred. The clinical course is shown in Figure 1G.

## Discussion

Therapeutic anti-PD-1/PD-L1 monoclonal antibodies, such as toripalimab, are important in treatments for patients with advanced NSCLC (Wang et al., 2023). We found that the sequential use of toripalimab and osimertinib was associated with SJS. Importantly, the toxicity appeared associated with toripalimab, given the fact that SJS has not recurred after osimertinib was rechallenged. The patient recovered after steroid treatment and benefited from the EGFR-TKI treatment that was followed. His OS was more than 44 months at the time of preparation the manuscript.

Skin reaction is one of the common adverse reactions of immune checkpoint inhibitors, and once it occurs, it needs to be discontinued permanently. However, with prednisone pre-treatment before using docetaxel, the probability of severe skin adverse reactions is very low, and delayed skin reaction after 1 week of drug use is rare. Based on previous clinical experience and other case reports, it is considered that the patient’s SJS is an adverse reaction to immune checkpoint inhibitors rather than to docetaxel. The incidence of SJS was low, but the lethality was extremely high. Although uncommon, SJS related to anti-PD1 in NSCLC has also been reported, such as pembrolizumab (Saw et al., 2017), atezolizumab (Chirasuthat and Chayavichitsilp, 2018), and ipilimumab (Dika et al., 2017). It has been reported that tumor tissues in NSCLC and skin shared similar antigens; thus, in patients treated with PD-1/PD-L1 antibodies, activated T cells may attack skin tissues as well, causing skin-related immune-related adverse events (irAEs) (Berner et al., 2019). The underlying mechanism of Stevens–Johnson syndrome/toxic epidermal necrolysis (TEN) associated with PD-1/PD-L1 and other drugs may be different. It is hypothesized that small-molecule drugs may bind to proteins in the serum, forming a complex that is recognized by certain HLA molecules and presented to T cells to generate an immune response (Frantz et al., 2021). However, in patients treated with PD-1/PD-L1 antibodies, the immune response is enhanced by the blockade of PD-1 and PD-L1 interaction instead of directly presenting PD-1/PD-L1 antibodies to T cells.

SJS could occur from 1 week to 5 months after the initiation of ICIs, which was usually 1–2 cycles of treatment (Chen et al., 2018). In our case, SJS started to manifest on day 12 since the start of toripalimab plus docetaxel administration. NGS detected HLA-A*24:02, HLA-A*11:01, HLA-B*40:01, HLA-B*15:01, HLA-C*04:01, and HLA-C*03:04 in this case. Because associations between SJS/TEN and certain human leukocyte antigen (HLA) variants have been identified, molecular diagnosis can help to confirm the diagnosis of SJS/TEN. A meta-analysis of Chinese, Korean, and Thai populations found HLA-A*24:02 associated with the susceptibility to SJS/TEN or mild maculopapular eruptions as lamotrigine-induced cutaneous adverse drug reactions (Deng et al., 2018). A study in the Japanese population also identified significant associations between HLA-A*24:02:01 and susceptibility to cold medicine-related SJS/TEN with severe ocular complications (Nakatani et al., 2019). It is possible that the HLA-A*24:02 allele in our patient conferred susceptibility to SJS upon treatment with toripalimab combined with docetaxel.

Toripalimab is a humanized monoclonal antibody and the first domestically approved anti-PD-1 monoclonal antibody in China. The pharmacokinetic (PK) characteristics of toripalimab within the dose range of 1–10 mg/kg showed that Cmax exhibited generally linear PK characteristics, and the increase in area under the curve was slightly greater than the increase in the dosage. The mean clearance rate of toripalimab was 0.18 mL/h/kg (co-efficient of variation%: 37%), and the geometric mean elimination half-life (t1/2) was 12.6 days (co-efficient of variation %: 29%). Toripalimab was degraded through non-specific pathways, and its metabolism was independent of clearance. As monoclonal antibodies are not metabolized by cytochrome P450 enzymes or other drug-metabolizing enzymes, the inhibition or induction of these enzymes by concomitant drugs is not expected to affect the PK of toripalimab. Docetaxel is a taxane that can form a stable, non-functional microtubule bundle by strengthening microtubule polymerization and inhibiting microtubule depolymerization, thereby breaking down tumor cell mitosis to achieve an antitumor effect. Clinical pharmacologic studies have confirmed that docetaxel’s antitumor activity is stronger than that of paclitaxel, and there is no cross-resistance with paclitaxel. It is used for the treatment of advanced or metastatic NSCLC after first-line chemotherapy failure. Chemotherapy in combination with immunotherapy has become one of the standard treatment regimens for lung cancer. Many phase III clinical studies of immunotherapy checkpoint inhibitors in the field of lung cancer have adopted paclitaxel in combination with platinum as the basis of chemotherapy regimen. Therefore, there are high-level safety data and evidence-based medical evidence for the combination of docetaxel and PD-1 inhibitors. Considering the patient’s economic burden, the relatively low-priced toripalimab was selected as the second-line treatment among the available immunotherapy checkpoint inhibitors.

Management principles of SJS include urgent inpatient evaluation/specialist support, prognostication with tools such as SCORTEN, withdrawal of culprit drug, and supportive care (Saw et al., 2017). The ideal management of severe anti-PD-1-related skin toxicities needs to be clarified. Intravenous prednisone/methylprednisolone 1–2 mg/kg/day and intravenous immunoglobulin are necessary.

The concomitant *EGFR* T790M/C797S in *trans* and *cis* is rare, with a poor prognosis (Liu et al., 2019). Previous studies showed that patients harboring EGFR C797S in *trans* with T790M are sensitive to a combination of first- and third-generation EGFR TKIs (Wang et al., 2017). However, patients harboring *EGFR* C797S in *cis* with T790M are resistant to combination therapy or every single reagent. In our case, the patient was sensitive to the combination of gefitinib and osimertinib, and the PFS was 8 m, indicating that gefitinib plus osimertinib might be an effective therapy for patients with *EGFR* T790M/C797S in *trans* and *cis*.

In the present case report, we provided timely treatment and discontinued the use of toripalimab when the diagnosis of SJS was made. However, we cannot fully exclude the possibility that SJS was caused by the administration of docetaxel. In the future, clinical usage of PD-1/PD-L1 antibodies in NSCLC patients harboring EGFR mutations needs to be cautious, and close attention should be paid to identify potential severe adverse events.

## Conclusion

We reported the first case of SJS induced by toripalimab in a patient with lung adenocarcinoma harboring multiple *EGFR* mutations, and the TKI treatment after SJS was well tolerated and effective. Our case report gave additional cautions to observe possible life-threatening cutaneous reactions to toripalimab therapy in NSCLC patients with *EGFR* mutations.

## Data Availability

The original contributions presented in the study are included in the article; further inquiries can be directed to the corresponding author.
