# Supplementary material for: Stevens–Johnson syndrome induced by toripalimab in a previously EGFR-TKI-treated advanced lung adenocarcinoma patient harboring EGFR mutations 19 del/T790M/C797S in trans and cis: a case report
Source: Front Pharmacol. 2023 Nov 14;14:1131703. doi: 10.3389/fphar.2023.1131703 (PMC10682071; doi:10.3389/fphar.2023.1131703)
Supplement: Supplementary file 1 [file Table1.docx]

**Supplementary Table S1. Patient's SCORTEN score**

| **Parameter** | **Value for SCORTEN (1 point)** | **Score of the patient** |
| --- | --- | --- |
| Age | ＞40 years | 1 |
| Cancer, hemopathy | yes | 1 |
| Percentage of skin detachment | ＞10% | 1 |
| Pulse rate | ＞120/min | 1 |
| Bicarbonaes | ＜20 mmol/L | 0 |
| Urea | ＞10 mmol/L | 0 |
| Glycemia | ＞14 mmol/L | 0 |
